# Ensemble Deep Learning Models on Raw DNA Sequences for Viral Genome Identification in Human Samples

**DOI:** 10.3390/s26072238

**Published:** 2026-04-04

**Authors:** Marco De Nat, Simone Boscolo, Sonia Pilar Gallo, Loris Nanni, Daniel Fusaro

**Affiliations:** Department of Information Engineering, University of Padova, 35131 Padova, Italysimone.boscolo.2@studenti.unipd.it (S.B.); soniapilar.gallo@studenti.unipd.it (S.P.G.); danielfusaro2@gmail.com (D.F.)

**Keywords:** neural networks, viroma, DNA sequence, ensemble

## Abstract

Detecting highly divergent or previously unknown viruses is a critical bottleneck in clinical diagnostics and pathogen surveillance. While alignment-based methods often fail to classify sequences lacking homology to known references, deep learning offers a powerful alternative for signal extraction from ‘viral dark matter.’ In this work, we present a high-performance ensemble of deep convolutional neural networks specifically designed to identify viral contigs in complex human metagenomic datasets. Our framework processes sequences acquired from high-throughput biological sensors and integrates complementary architectures to capture both local motifs and global genomic signatures. The proposed ensemble achieves state-of-the-art performance, reaching an AUROC of 0.939 on 300 bp contigs and significantly outperforming existing models such as transformer-based approaches, ViraMiner, and DeepVirFinder. Crucially, our results demonstrate high robustness to data degradation, maintaining stable predictive power even with a 10% random nucleotide substitution rate, a common challenge in degraded clinical samples. Furthermore, the model generalizes to ‘unseen’ viral families not present during training, demonstrating its utility for emerging threat detection. To ensure full reproducibility and facilitate further research in clinical sensing, the complete code and datasets are publicly available on Github.

## 1. Introduction

DNA sequences lie at the core of all living systems, shaping virtually every biological process in both eukaryotes and prokaryotes. Understanding how information is encoded in DNA, and how sequence properties give rise to biological function, is fundamental not only for advancing basic biology but also for engineering new biological systems. However, the immense diversity and complexity of possible DNA sequences make such tasks inherently challenging. Many coding and noncoding regions remain poorly characterized, and traditional analytical methods often struggle to capture the full breadth of patterns embedded in biological data [1]. The accurate classification of viral contigs from metagenomic next-generation sequencing (mNGS) data has emerged as a cornerstone of modern clinical diagnostics and public health surveillance [2,3]. In clinical settings, the unbiased nature of mNGS allows for the detection of viral pathogens in cases of idiopathic infections where traditional targeted assays, such as PCR, often yield inconclusive results [4]. Furthermore, in the context of global health surveillance, rapid and robust viral identification is essential for the early detection of emerging zoonotic threats and the characterization of ‘viral dark matter’, i.e., divergent viral sequences that lack close homologs in existing databases [5]. By effectively filtering viral signals from the overwhelming background of host and bacterial DNA, automated classification tools enable real-time genomic epidemiology, which is critical for monitoring outbreak dynamics and informing pandemic preparedness [6].

Machine learning has therefore become indispensable in bioinformatics, enabling the extraction of meaningful insights from rapidly expanding datasets. Classical methods such as random forests, support vector machines, hidden Markov models, and Bayesian networks have long contributed to fields including genomics, proteomics, systems biology, and structural biology [7]. More recently, deep learning has surpassed these traditional approaches by scaling to massive datasets and uncovering complex, nonlinear relationships. The transformative impact of deep learning is exemplified by breakthroughs such as AlphaFold 3 [8] and by the growing use of natural language processing-inspired models to analyze biological sequences, which resemble linguistic data in their structure and statistical properties [9]. While deep neural networks are often characterized as ‘black boxes,’ their ability to recognize complex, nonlinear patterns serves as a critical first step in the decoding of the DNA program. By identifying distinctive viral motifs across diverse genomic contexts, these models provide the raw material for future functional studies and biological interpretation [10].

These advances are particularly relevant to the study of the human virome, the diverse community of viruses that inhabit the human body. Virome composition varies across individuals and has been linked to disease, yet its full influence on human health remains incompletely understood [11,12]. Metagenomic studies continue to reveal large numbers of previously unknown viruses, suggesting that only a fraction of human associated viruses have been discovered. Emerging evidence further indicates that yet unidentified viral pathogens may contribute to complex conditions [13], including autoimmune diseases such as diabetes [14] and multiple sclerosis [11,15]. Conversely, the study of the human virome also reveals viruses with potential beneficial roles, such as oncolytic viruses that can be engineered or naturally occur to target and eliminate malignant cells, offering new frontiers in anticancer therapy [16,17].

Next-generation sequencing (NGS) platforms act as sophisticated biological sensors that convert biochemical events into digital signals through high-resolution optical or electrical transducers. NGS has become a powerful means of directly capturing all genetic material present in clinical samples, enabling metagenomic analyses that bypass the need for prior knowledge of pathogens [18]. However, current computational approaches face significant limitations. Alignment-based tools such as BLAST 2.17.0 often fail to classify highly divergent sequences, leaving many reads labeled as “unknown”. Profile hidden Markov model approaches like HMMER3 [19] improve sensitivity to distant homologs but still depend on existing reference databases, restricting their ability to detect highly novel viral genomes [15]. Integrating modern machine learning and deep learning techniques with metagenomic sequencing presents a crucial opportunity to advance viral discovery and deepen our understanding of the human virome [7,15].

The clinical motivation for this work stems from the urgent need to identify viral pathogens in complex metagenomic samples where traditional alignment-based methods, such as BLAST, often reach their functional limits. In clinical diagnostics, two primary challenges arise: evolutionary divergence and data degradation.

First, emerging or rapidly evolving viruses often reside in the homology twilight zone, where sequence identity to known references is too low for standard tools to trigger a match. Our ensemble model addresses this by learning deep compositional patterns, enabling the detection of previously unseen viruses, albeit with moderate performance, and thereby contributing to the exploration of viral dark matter.

Second, clinical samples can be subjected to degradation or sequenced using high-throughput but error-prone technologies. By demonstrating State-of-the-Art (SOTA) performance even when faced with 10% random nucleotide noise, our framework ensures diagnostic robustness in scenarios where sequence integrity is compromised.

The main contributions of this work can be summarized as follows:

- We introduce an ensemble of neural networks that leverages heterogeneous feature representations for training, improving robustness and generalization.
- The proposed approach is built upon a well-established, for this topic, neural network architecture, extending it through the exploration of different network hyperparameters and pooling strategies to construct the ensemble.
- We provide a publicly available implementation of the proposed system to ensure reproducibility https://github.com/LorisNanni/Ensemble-Deep-Learning-Models-on-Raw-DNA-Sequences-for-Viral-Genome-Identification-in-Human-Samples (accessed on 1 April 2026).
- Extensive experimental evaluations demonstrate that the proposed method achieves state-of-the-art performance when compared to existing approaches, such as [7,15,20,21], on the same datasets and under the same evaluation protocols. In particular, we used different datasets with contigs of varying lengths. We evaluated multiple levels of noise to validate the robustness of our method, and we also conducted a test using an unseen test set in which the species did not overlap with those in the training set.

We acknowledge that a wide range of neural network architectures exists, as well as numerous methods for representing DNA sequences as matrices suitable for neural network processing [22]. However, providing an exhaustive survey or comparison of these innumerable approaches is not the objective of this work. Instead, our goal is to demonstrate that a simple ensemble of neural networks, composed of small models and therefore characterized by low computational cost on modern GPUs, is sufficient to achieve state-of-the-art performance on the evaluated datasets and task. Importantly, this is accomplished without making any a priori hyperparameter choices based on the particular test dataset, providing a single ensemble model architecture and thereby avoiding potential sources of overfitting. All design decisions involved in constructing the ensemble are derived exclusively from the training/validation set, ensuring a principled and unbiased evaluation protocol.

## 2. Materials and Methods

The proposed pipeline is conceptualized as an integrated sensing and interpretation system. The data acquisition phase relies on NGS optical sensing, which monitors fluorescence-labeled nucleotide integration to produce reads. Our ensemble model serves as the signal processing unit, achieveing robust classification performance even when the sensing accuracy decreases and maintaining high reliability up to a 10% simulated noise level in the data acquisition.

In this section, we describe the neural network architectures adopted in this study, with particular emphasis on the ViraMiner architecture [15], which serves as the baseline model for viral sequence identification. We also detail the preprocessing strategy used to transform raw DNA sequences into a numerical representation suitable for convolutional neural networks.

All DNA sequences are encoded using the well-established one-hot encoding scheme for nucleotide sequences [23]. Let

A={A,C,G,T,N}

denote the alphabet of possible characters, where (A), (C), (G), and (T) represent the four canonical nucleotides, and (N) is used as a fifth symbol to encode any ambiguous or non-canonical nucleotide. Each nucleotide (x∈A) is mapped to a binary vector ($v\left(x\right)\in {\left\{0,1\right\}}^{5}$), defined as $v{\left(x\right)}_{i}\;=\;\left\{\begin{array}{cc} 1, & \mathrm{if}\;x\;=\;A_{i}\\ 0, & \mathrm{otherwise}. \end{array}\right.$

**Figure 1:**
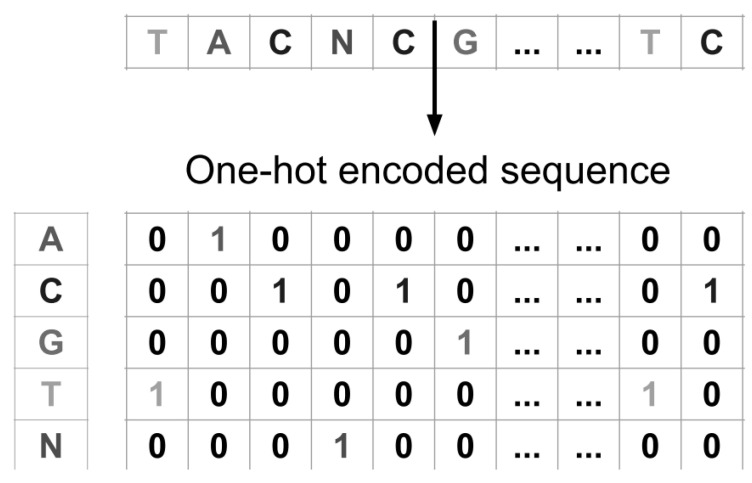
One hot encoding process visualization of a sample DNA sequence.

Given a DNA sequence of fixed length *L*, $S=(x_{1},x_{2},\ldots ,x_{L})$, the one-hot encoding transforms (*S*) into a binary matrix $X\in {\left\{0,1\right\}}^{L\times 5}$ where each row corresponds to the one-hot representation of a single nucleotide in the sequence. This matrix constitutes the input to the neural networks described in the following. See Figure 1 for an example of one hot encoding.

**Figure 2:**
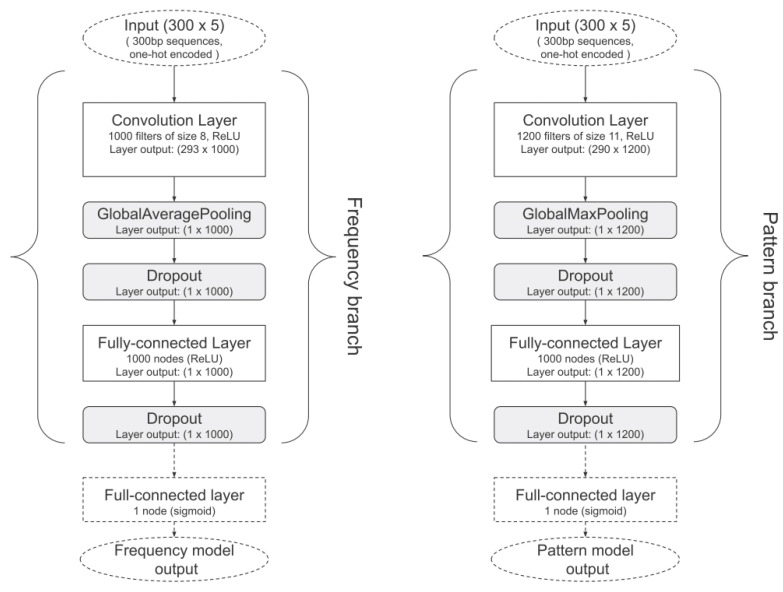
Original ViraMiner branches. In our modified version, we added a BN step after the convolutional layer and after the first fully connected layer.

**Figure 3:**
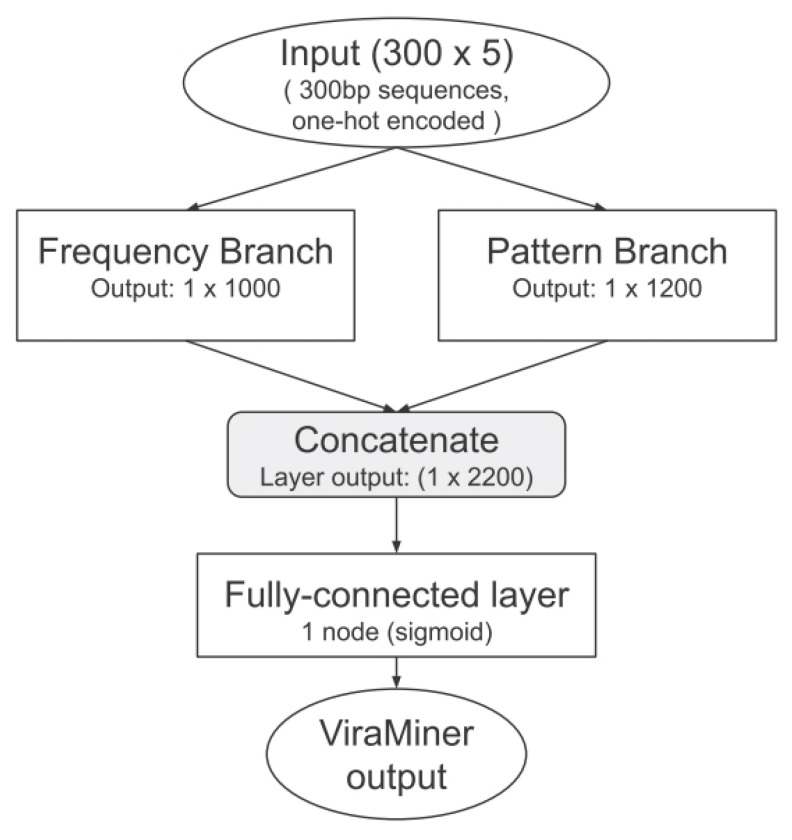
ViraMiner full architecture.

Our baseline is ViraMiner, a convolutional neural network (CNN)-based method designed for the identification of viral sequences integrated into the human genome. This choice is well motivated, as CNNs have already proven effective in analyzing biological sequences, such as DNA and amino acid sequences [24,25,26]. The architectures evaluated in this study are derived from the models illustrated in Figure 2 and Figure 3. Input DNA sequences are first encoded using a fixed-length numerical representation and processed by multiple parallel convolutional branches that operate at different receptive field sizes, enabling the model to capture both short-range and long-range sequence patterns. The convolutional layers perform hierarchical feature extraction, while pooling layers progressively reduce the dimensionality and improve translation invariance. The extracted features are then flattened and passed to fully connected layers that act as the final classifier. The network outputs a single sigmoid-activated probability score, indicating the likelihood that the input sequence contains viral genomic material. Moreover, we added a batch normalization (BN) step after the convolutional layer and after the first fully connected layer.

The model is trained using the Adam algorithm [27] to optimize the binary cross-entropy loss function.

In addition to the branches shown in Figure 2, we also designed another branch, called the LP branch, in which the pooling operator is a parameterized pooling, whose parameter is learned during training. The parameterized pooling, named “Power-Average” pooling [28], is defined per-window as follows:

(1) $$y={\left(\frac{1}{N}\sum_{i=1}^{N}x_{i}^{p}\right)}^{\frac{1}{p}}.$$

when p=∞, this corresponds to Max Pooling; *p* = 1 corresponds to Mean Pooling. In the context of Power-Average Pooling in Table 1, the power hyperparameter *p* is referred to as ‘Norm type’.

The hyperparameters tested over all the models are reported in Table 1. It is important to emphasize that all hyperparameters were selected using only the validation set. The test set is completely blind and was never used for hyperparameter tuning to avoid any form of overfitting on our data and to ensure that the reported results are as reliable as possible. As the learning-rate scheduler, we used a decay approach, which reduces the optimizer’s learning rate by a factor of 0.5 every 5 epochs.

Throughout the remainder of the paper, the term branch refers to the network associated with a specific backbone (e.g., columns in Figure 2). For instance, the branch “pattern” corresponds to the network shown in the last column of Figure 2.

**Figure 4:**
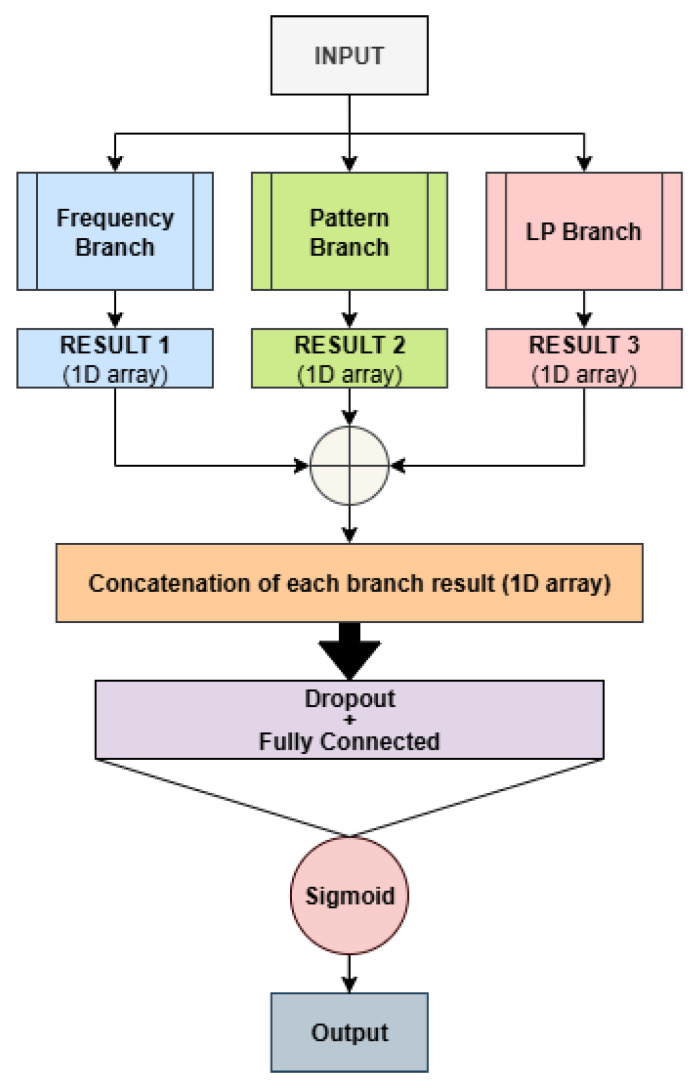
Our best model architecture, integrating all three proposed branches.

The merger combines multiple branches into a single system, as illustrated in Figure 4. Specifically, the outputs of the first fully connected layer of all branches are concatenated, followed by a dropout layer and a fully connected layer that produces the final model output. For the merger models, only the best-performing branch of each type was included, using the hyperparameters selected for each branch when trained independently.

The ensemble proposed in this work consists of ten networks based on the previously presented architectures. When the architecture name starts with B_*x*_, it denotes a single-branch network of type *x*, as illustrated in the previous Figure 2, where x=f corresponds to the frequency branch, x=p to the pattern branch, and x=l to the LP branch. Conversely, architectures labeled M_*x*_+_*y*_ indicate multi-branch models that combine branches *x* and *y*, as exemplified in Figure 4. The ensemble is constructed using a simple sum rule, where the outputs of the individual networks are fused by summation, without introducing any additional learnable parameters.

When the term ‘VM’ appears in parentheses after the network name, it indicates that we use exactly the same architectures as those reported in [15], i.e., identical layers and hyperparameters. For example, B_*f*_ (VM) indicates that we use exactly the same structure and topology as the branch frequency neural network proposed in ViraMiner. If, instead, it is labeled simply as B_*f*_, then we use the architecture described above.

In conclusion, the proposed ensemble is composed of the following networks:

- B_*f*_ (VM);
- B_*f*_;
- B_*l*_;
- B_*p*_ (VM);
- B_*p*_;
- M_*f*_+_*p*_+_*l*_;
- M_*f*_+_*l*_;
- M_*p*_+_*l*_;
- M_*f*_+_*p*_;
- Original ViraMiner architecture.

Please note that for the merger architecture, we use only the branches proposed here, and not the original ones from ViraMiner.

As can be seen from the networks that make up the ensemble, our approach to constructing it is fairly straightforward. We introduced several architectural modifications to the network: we added layers to the branches proposed in ViraMiner, as explained earlier; we created a new branch using a different pooling method; and we designed a new merger that combines three branches, since our approach uses three branches, whereas the original ViraMiner employs only two. Despite its relative simplicity, using slightly different architectures allowed us to build an ensemble that achieves better performance than the state of the art.

### 2.1. Datasets

In this study, we used multiple datasets. The first, called VM, is the one commonly used in the literature and was originally proposed by the authors of ViraMiner [15]. We then used an “Unseen” dataset, in which the species present in the test set do not appear in the training set. We obtained this dataset as a subset of VM by removing all instances of a particular viral class from both the training and validation sets. We then constructed a test set consisting only of instances from that viral class, along with all non-viral instances.

Additionally, we generated a simulated noisy dataset (Noise) based on VM dataset to further validate our results and a simulated dataset (SIM) generated with the ART sequencing read simulator [15].

Finally, we included a dataset, named LongC, where each virus is represented by a long sequence of 3000 contigs, as proposed by the authors of DeepVirFinder [20]. In Table 2, the sizes of the training, validation, and test sets for all evaluated datasets are reported.

#### 2.1.1. VM Dataset [15]

The metagenomic data used in this study were obtained through NGS technologies, including Illumina NextSeq, MiSeq, and HiSeq platforms, following the manufacturers’ standard protocols. The dataset originated from human-derived samples collected from multiple patient cohorts. These analyses aimed to identify viral genomes and other microorganisms in individuals affected by disease, as well as in matched control subjects.

All sequencing runs were processed using a validated bioinformatics pipeline. In brief, the workflow begins with read quality assessment, during which sequences are filtered according to their Phred quality scores. High-quality reads are then aligned against human, bacterial, phage, and vector reference genomes using BWA-MEM, and any reads showing more than 95% sequence identity over at least 75% of their length are excluded from subsequent analyses [29]. The remaining reads are normalized and assembled de novo using multiple assemblers, including IDBA-UD, Trinity, SOAPdenovo, and SOAPdenovo-Trans. The resulting contigs undergo taxonomic assignment via BLAST. The implementation of the pipeline is publicly available on GitHub (https://github.com/NGSeq/ViraPipe (accessed on 1 April 2026) and https://github.com/NIASC/VirusMeta (accessed on 1 April 2026)).

The dataset comprised 19 independent NGS experiments originating from human sample types such as skin, serum, and condylomata. After de novo assembly, contigs were annotated using PCJ-BLAST [30] with the following configuration: Blastn algorithm, match reward of 1, mismatch penalty of 1, gap opening cost of 0, gap extension cost of 2, and an e-value threshold of $1\times 10^{-4}$. Therefore the labels are assigned using PCJ-BLAST. Notice that a contig is a continuous DNA sequence assembled from overlapping shorter sequencing reads. To prepare the input data for neural networks, labeled contigs were segmented into fixed-length fragments of 300 bp; any residual nucleotides that did not fit into a full-length segment were discarded. The dataset contains around 320,000 DNA sequences, considering the discarded ones, overall. Its primary difficulty lies in the significant class imbalance, as viral sequences make up only about 2% of the total. The data was divided into training, validation, and test subsets. The most common viral classes are listed in the following:

- Anelloviridae;
- Caudovirales;
- Geminiviridae;
- Genomoviridae;
- Herpesvirales;
- Inoviridae;
- Circoviridae;
- Iridoviridae;
- Microviridae;
- Mimiviridae;
- Papillomaviridae;
- Parvoviridae;
- Phycodnaviridae;
- Polyomaviridae;
- Poxviridae;
- Retroviridae.

#### 2.1.2. Unseen

Training and validation datasets are derived from the VM dataset, with all anellovirus sequences removed from both sets. The test set is composed of both non-viral sequences (26,296 contigs) and anellovirus sequences (1348 contigs). In this setting, anelloviruses represent a completely novel viral category from the model’s perspective, as they are not encountered during training. This setup enables evaluation of the model’s capability to identify viruses belonging to entirely unseen classes. The sequence processing and labeling steps are identical to those used for the VM dataset.

#### 2.1.3. SIM

This dataset is generated with the ART sequencing read simulator [31] (see [15] for details on simulation settings). The simulated dataset contained slightly fewer reads than the real one (210,000) but included a higher proportion of viral sequences (around 10%).

#### 2.1.4. Noise

The training and validation datasets are identical to those of the original VM dataset. We generated three different test sets from the original VM test set by introducing random point mutations (substitutions) at rates of 1%, 5%, and 10%. For each mutation rate, we created five distinct versions of the test set using different seeds for the pseudo-random number generator, enabling statistical analysis. These generated datasets are available in the GitHub repository for this work.

Specifically, for each sequence of length (*N*), we randomly selected (*p* · *N*) unique positions (without replacement), where (*p* ∈ 0.01, 0.05, 0.10). At each selected position, the original nucleotide was substituted with one of the remaining bases. This procedure ensures that mutations are independently and uniformly distributed across the sequence, with no positional bias or clustering effects.

Importantly, the 10% noise level represents an intentionally extreme scenario. Sequencing technologies, such as those developed by Oxford Nanopore Technologies, typically have empirical error profiles that are lower and, crucially, not uniformly random. Real sequencing errors tend to exhibit structured patterns (e.g., context-dependent substitutions, insertions/deletions, or localized error clusters), rather than the independent and identically distributed substitutions assumed here. As a result, our 10% uniformly random noise configuration can be interpreted as a worst-case upper bound, where errors are maximally dispersed and thus more difficult to model or correct.

By adopting this simplified and adversarial noise model, we aim to stress-test the robustness of the proposed method under conditions that are more challenging than those encountered in realistic sequencing scenarios.

#### 2.1.5. LongC [20]

The dataset is constructed from viral and prokaryotic genomes obtained from RefSeq, partitioned temporally into training, validation, and test sets to avoid overlap; see [20] for details. Genomes are fragmented into non-overlapping sequences to mimic real metagenomic contigs, with a balanced number of viral and non-viral sequences.

## 3. Results

### 3.1. Performance Indicators

The Area Under the Receiver Operating Characteristic curve (AUROC), Precision, and Recall were used as evaluation metrics to identify the best-performing models. For a binary classification problem with true positives (TP), false positives (FP), true negatives (TN), and false negatives (FN), the metrics are defined as follows:

- $\mathrm{Precision}=\frac{TP}{TP+FP}$;
- $\mathrm{Recall}=\frac{TP}{TP+FN}$;
- The ROC curve plots the True Positive Rate (TPR) against the False Positive Rate (FPR):
  $\mathrm{TPR}=\frac{TP}{TP+FN},\;\mathrm{FPR}=\frac{FP}{FP+TN}$.
  The AUROC is the area under this curve: $\mathrm{AUROC}=\int_{0}^{1}\mathrm{TPR}\left(\mathrm{FPR}\right)d\left(\mathrm{FPR}\right)$

These performance indicators are also used in the literature, since accuracy is not informative when the dataset is highly imbalanced and, as is well known in the literature, accuracy is not a reliable metric for strongly imbalanced datasets.

To assess the statistical reliability and stability of the classification performance, we estimated the 95% Confidence Intervals (CI) [32] for the AUROC curve using a nonparametric bootstrap approach. The method involves generating N=1000 resampled datasets with replacement from the original test set, maintaining the same sample size. For each bootstrap iteration, the AUROC is recalculated to build an empirical distribution of the metric. The 95% confidence interval was calculated as $\left[Q_{0.025},Q_{0.975}\right]$, where *Q* represents the quantiles of the bootstrap distribution. This approach provides a robust measure of uncertainty without assuming a normal distribution of the performance scores, ensuring that the reported improvements over baseline models are statistically significant and not due to specific test set composition.

DeLong’s method [33] provides a nonparametric test for comparing ROC curves by evaluating differences in their areas under the curve. Unlike a simple assessment of confidence interval overlap, the DeLong test estimates the variance and covariance of the AUCs using a U-statistics framework.

### 3.2. Results on VM Dataset

In Table 3, we report the following approaches in the VM dataset:

- FullE, the ensemble proposed in this work;
- M_*f*_+_*p*_+_*l*_, our best stand-alone net;
- Viraminer (VIR), the original method proposed in [15];
- DeepVirFinder (DVF), the original method proposed in [20];
- 6-mers, 6-mer frequency features [34] used for feeding a Random Forest classifier, The AUROC values for 3-, 4-, 5-, 6- and 7-mers were 0.867, 0.872, 0.873, 0.875 and 0.869, respectively; 6-mers get the highest AUROC.
- Ref. [7]. The comparison of our method with this paper is very interesting, not only because it uses the same dataset and the same data split, but also because this method is based on self-attention and convolutional operations on nucleic acid sequences, leveraging two prominent deep learning strategies commonly used in computer vision and natural language processing.

As shown in Table 3, our method achieves the best performance in terms of AUROC. Using the DeLong method to compare the different approaches, we can reject the null hypothesis that two methods yield the same AUROC with a *p*-value of 0.01 for all methods, except for a *p*-value of 0.05 when compared against [7].

The trade-off between precision and recall is a critical aspect of viral genome classification. A contig is labeled as viral when the model output exceeds a specified threshold. In [15], the authors report achieving a precision of 0.90 with a recall of 0.32. By contrast, when our ensemble model is tuned to the same precision level (0.90), it attains a higher recall of 0.4011.

**Figure 5:**
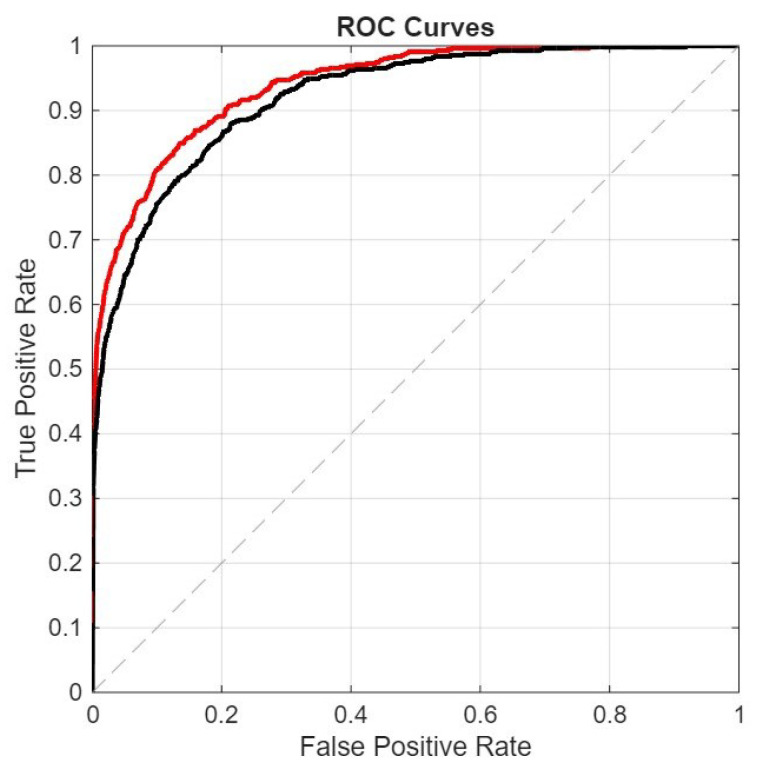
ROC curves: proposed ensemble (red curve) vs. original ViraMiner model (black curve).

**Figure 6:**
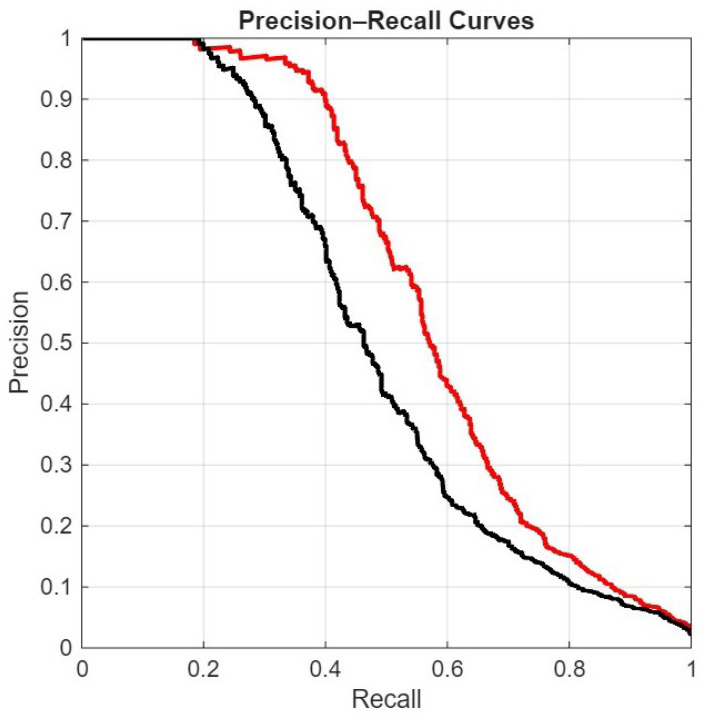
Precision–Recall curves: proposed ensemble (red curve) vs. original ViraMiner model (black curve).

**Figure 7:**
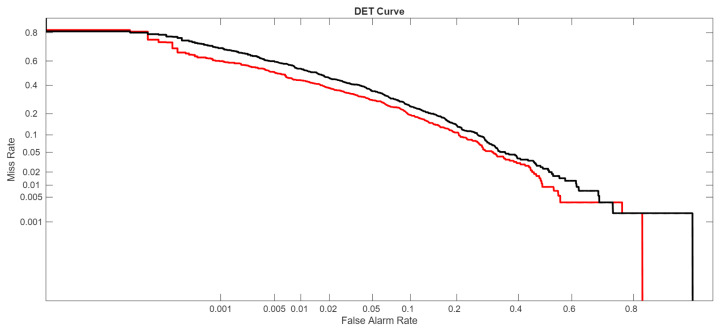
DET curves: proposed ensemble (red curve) vs. original ViraMiner model (black curve).

In the following figures, we directly compare our ensemble (red curve) with the original ViraMiner model (black curve): in Figure 5 using the ROC curve, in Figure 6 using the precision–recall curve, and in Figure 7 using the DET curve [35]. A Detection Error Trade-off (DET) curve plots the trade-off between Miss Rate (false negative rate) and False Alarm Rate (false positive rate) across varying decision thresholds. In these cases, the performance difference between the two methods is clear. It is evident that this performance gain does not come for free, since the inference time of our approach is roughly ten times higher than that of the original ViraMiner. However, we consider this a secondary issue given the computational power currently available with modern GPUs. The ensemble, evaluated on a Titan RTX (24 GB), classified 100k contigs in approximately 123 s. Notably, these results were obtained using a single GPU. While the Titan RTX represents a previous generation, it provides a conservative estimate of performance. Given that inference across the ensemble of networks is fully parallelizable, leveraging multiple GPUs or more recent hardware such as an NVIDIA GeForce RTX 5090 would likely yield a substantial reduction in inference time.

#### Principal Component Analysis on VM Dataset

**Figure 8:**
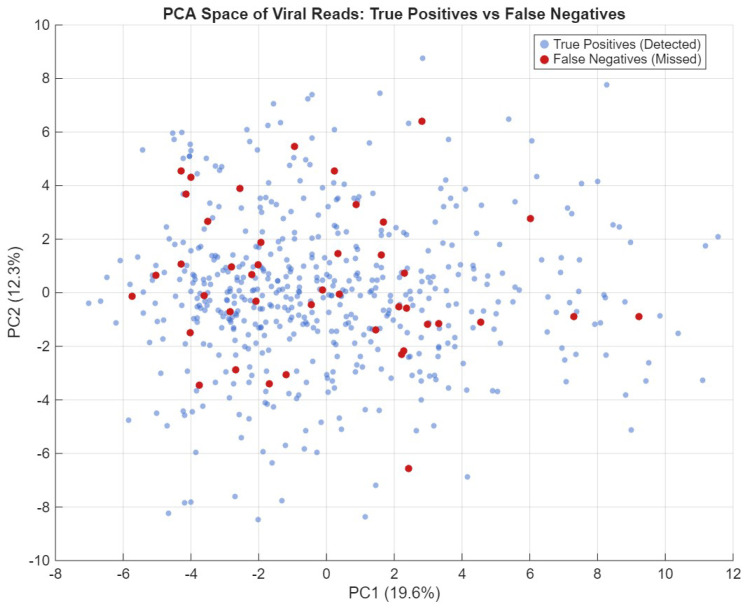
PCA projection: True Positives and False Negative patterns.

Principal Component Analysis (PCA) [36] is a widely used dimensionality reduction technique that preserves the main sources of variance in high-dimensional data, enabling exploratory analysis of class separability. To create the PCA plot, we represented each sequence using the 3-mer technique [37] and then applied principal component analysis to project the data into two dimensions. The PCA analysis of viral reads (Figure 8) shows that False Negatives are not clustered in a specific region of the sequence space. Instead, they are interspersed with True Positives. This suggests that the model’s misses are not due to a failure in recognizing specific viral families, but rather to the intrinsic noise of short 300 bp reads, where the viral signal is occasionally too weak to be distinguished from the genomic background.

### 3.3. Results on Noise Dataset

**Figure 9:**
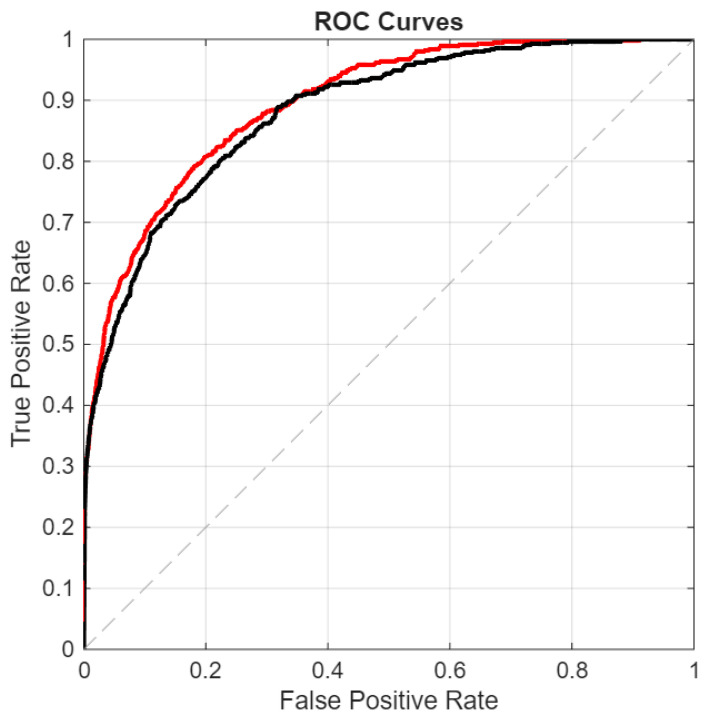
Noise-ROC curves: proposed ensemble (red curve) vs. original ViraMiner model (black curve).

**Figure 10:**
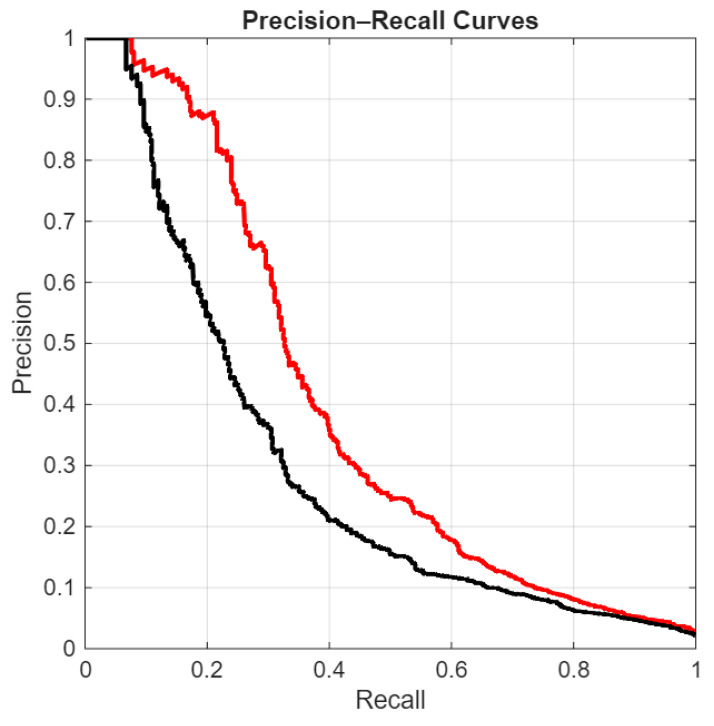
Noise Precision–Recall curves: proposed ensemble (red curve) vs. original ViraMiner model (black curve).

**Figure 11:**
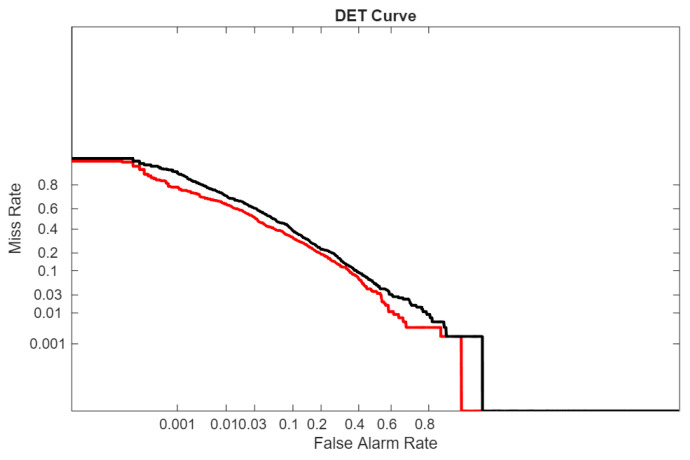
Noise-DET curves: proposed ensemble (red curve) vs. original ViraMiner model (black curve).

In Table 4 and in Figure 9, Figure 10 and Figure 11, we report the performance of our ensemble and our best method, comparing them with ViraMiner and DeepVirFinder on the noise dataset. It consists of three levels of perturbation of the original VM test set, with mutation rates of 1%, 5%, and 10%. For each level of perturbation, we generated five versions using different seeds for the pseudo-random number generator and report the mean performance in the following. This setup allows us to systematically evaluate model robustness under increasing degrees of sequence corruption.

Across all the tests, regardless of the noise level, our ensemble achieves superior performance. We can reject the null hypothesis with a *p*-value of 0.01, concluding that our ensemble does not achieve the same performance as DeepVirFinder or ViraMiner.

### 3.4. Results on the Unseen Dataset

**Figure 12:**
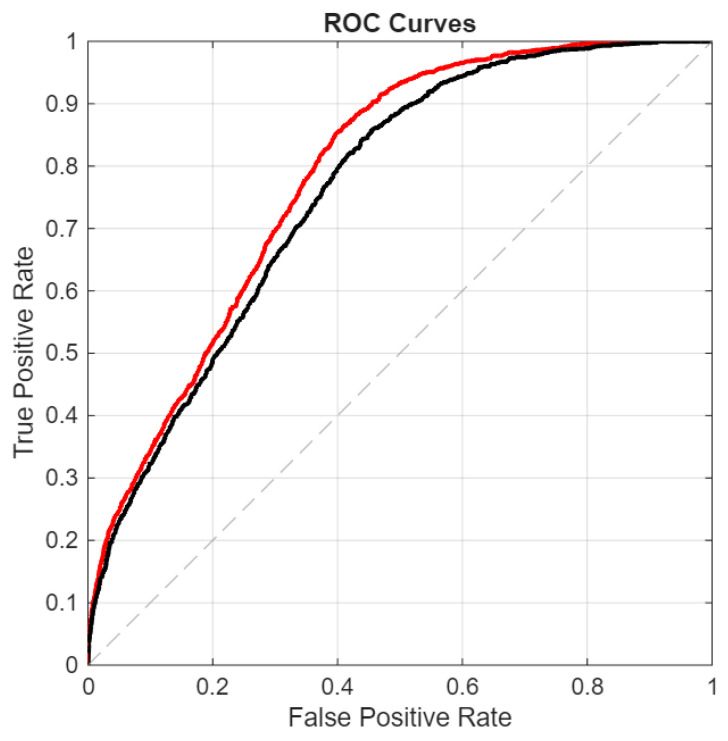
Unseen-ROC curves: proposed ensemble (red curve) vs. original ViraMiner model (black curve).

**Figure 13:**
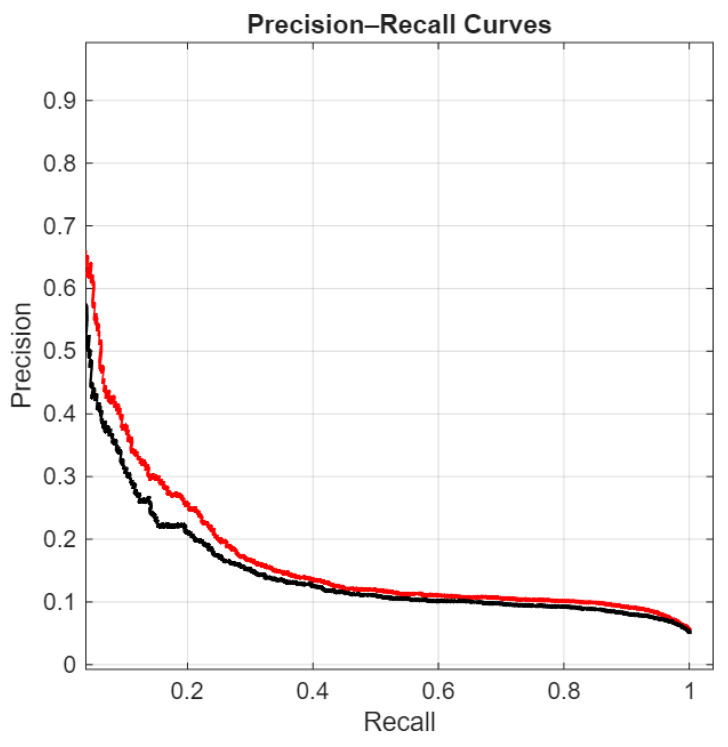
Unseen Precision–Recall curves: proposed ensemble (red curve) vs. original ViraMiner model (black curve).

**Figure 14:**
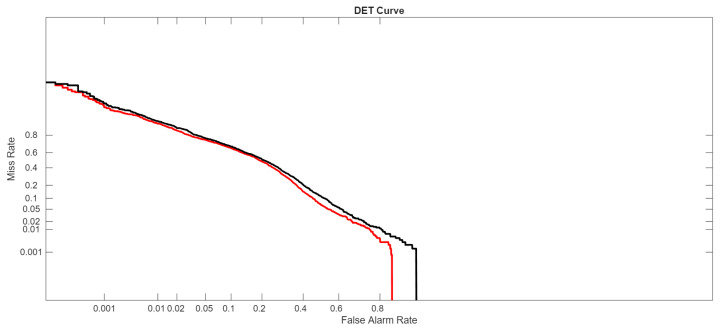
Unseen-DET curves: proposed ensemble (red curve) vs. original ViraMiner model (black curve).

To assess the model’s ability to generalize to previously unseen viral classes, we designed an experiment in which all Anellovirus sequences were excluded from both the training and validation datasets. Results are reported in Table 5 and in Figure 12, Figure 13 and Figure 14; consistent with the other tests, our ensemble achieves superior performance in this setting as well. We reject the null hypothesis (*p* = 0.01), concluding that its performance differs significantly from that of DeepVirFinder and ViraMiner. However, unlike the other datasets, in this test, the best proposed architecture achieves performance comparable to that of the full ensemble. What is important, however, is that the ensemble improves upon the baseline methods, ViraMiner and DeepVirFinder. As can be clearly observed, the area under the ROC curve is significantly lower than in previous tests; however, the task at hand is considerably more challenging. In this case, the goal is to recognize viral sequences belonging to species that were not included in the training set. Therefore, any improvement over the previous state of the art represents a result of substantial scientific relevance.

### 3.5. Results on the LongC Dataset

Results are reported in Table 6; consistent with the other tests, our ensemble also achieves superior performance in this setting. We reject the null hypothesis (*p* = 0.01), indicating that its performance differs significantly from that of DeepVirFinder and ViraMiner.

Moreover, performance on this dataset is substantially higher than on the previous one; for this reason, we do not report the curves, as they are all very close to a near-perfect result. Despite this, the proposed ensemble approaches even more closely an area under the ROC curve of 100, corresponding to an almost perfect discrimination between the classes in our dataset.

### 3.6. Further Comparisons

To ensure that the effectiveness of our model was not influenced by unnoticed biases in the real datasets, as in [15], we additionally evaluated it using SIM. We first applied the models trained exclusively on the 19 real metagenomic datasets: this produced an AUROC of 0.751 for Viraminer and 0.782 for our approach. Next, we retrained ViraMiner and our ensemble directly on the simulated data; we reused the same hyperparameters and training setup as before. The model was trained on 80% of the simulated data, with 10% for validation and 10% for testing. Under this configuration, the ViraMiner achieved a test AUROC of 0.921, while our approach achieved 0.932.

As a further experiment, we evaluated a foundation model for DNA barcoding as a feature extractor. The extracted features were subsequently used to train a Support Vector Machine (SVM), LibSVM toolbox (https://www.csie.ntu.edu.tw/~cjlin/libsvm/ (accessed on 1 April 2026)). This experiment is particularly interesting because the foundation model was pre-trained on DNA sequences that are completely different from those analyzed in this study. Specifically, we employed a masked language model (MLM), BarcodeMAE, as a foundation model to extract numerical vector representations (embeddings) from DNA sequences. BarcodeMAE was originally trained on arthropod DNA barcoding data, which is biologically unrelated to our target domain. Despite this domain mismatch, our goal was to investigate whether a model pre-trained on large-scale, heterogeneous genomic data could still capture generalizable sequence features useful for downstream tasks. To obtain a fixed-length representation of each DNA sequence, we derived a single embedding for the entire DNA sequence by applying global average pooling over the sequence of 768-dimensional token-level output vectors produced by the model, excluding padding and special tokens. These pooled embeddings were then used as input features to train the SVM classifier. As expected, the standalone performance of this approach was not particularly strong. The SVM trained solely on the embeddings extracted from the pre-trained MLM achieved an AUROC of 0.6818, indicating only moderate discriminative capability. Nevertheless, a more interesting finding emerged when combining this classifier with our main ensemble. By fusing the SVM outputs with the ensemble predictions using a simple weighted sum rule, where the weights are obtained using the validation data, we observed a slight improvement in overall performance, reaching an AUROC of 0.9395, which is marginally higher than that of the proposed ensemble alone. Although the gain is minimal, it is noteworthy, as it suggests that the foundation model still captures non-random and complementary information that can contribute to downstream classification.

**Figure 15:**
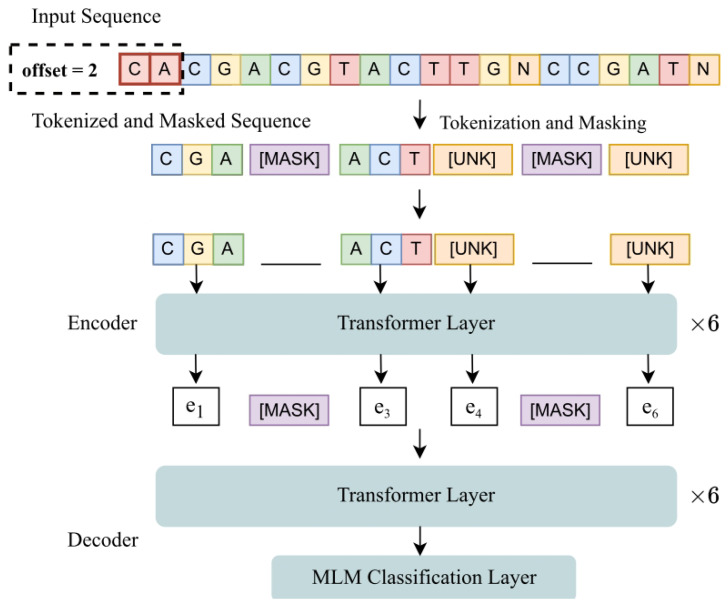
BarcodeMAE Architectures.

The architecture of the foundation model is shown in Figure 15.

As a final experiment, we evaluate the EDEN method [38], a multiscale DNA sequence encoding framework based on kernel density estimation. EDEN represents nucleotide sequences as position-aware density profiles computed at multiple biologically relevant scales and processes them with a hybrid deep convolutional neural network. We consider two evaluation settings: (i) training the EDEN architecture from scratch using our training set, and (ii) fine-tuning an EDEN model pre-trained shared by the original authors of EDEN (https://github.com/zabihis/EDEN (accessed on 1 April 2026)). When training EDEN network from scratch, performance is very low. It improves when using a pre-trained version. We conducted several experiments, also varying the optimization methods. The best method, consistently selected based on the validation set, achieves an area under the ROC curve (AUC) of 0.8775 on the test set, which is still clearly lower than the performance of the methods we propose. If we include this method in our ensemble, through a weighted fusion, where the EDEN weight is selected using the validation set (with the ensemble weight fixed to 1), we obtain an AUC of 0.9395. If, within the same weighted combination, we also add the scores produced by an SVM trained on Barcodemae, with its weight again determined on the validation set, the AUC further increases to 0.9399. Although these improvements are modest and mainly refine the performance, it is interesting to note that feature extraction methods fully pre-trained on datasets different from the one used in our study can still contribute to enhancing overall performance. Given the greater complexity of this ensemble, it is not our recommended approach; instead, we favor the previously proposed ensemble. Nevertheless, the results remain noteworthy. On one hand, EDEN clearly does not achieve performance comparable to our method. This suggests that simply using one of its pre-trained versions and fine-tuning it on our dataset is not sufficient to obtain state-of-the-art results. On the other hand, it is interesting to observe that features extracted from a foundation model trained for insect DNA barcoding can still be leveraged to train an SVM that performs non-random classification on the problem addressed in this work, albeit with relatively low performance.

### 3.7. The Role of the Ensemble Framework in Metagenomic Pipelines

The proposed ensemble framework is designed to function as a high-performance signal processing unit within clinical and environmental metagenomic pipelines. In this context, next-generation sequencing platforms act as primary biological sensors, where biochemical nucleotide incorporation events are transduced into digital information. Our tool occupies a critical position in the post-acquisition stage, acting as an intelligent filter that distinguishes the “viral signal” from the overwhelming “genomic noise” of the host and bacterial background. A primary challenge in metagenomic sensing is the detection of sequences within the “homology twilight zone” viral fragments that lack significant sequence identity to known references. While traditional alignment-based tools, such as BLAST, rely on $S_{similarity}\;>\;\theta$ (where θ is a high stringency threshold), our model learns deep compositional signatures, enabling the identification of unseen viruses. By maintaining an AUROC ≈ 0.89 under 10% noise conditions or AUROC ≈ 0.79 with unseen virus, the tool ensures diagnostic robustness where standard pipelines typically fail. Beyond simple classification, this pattern recognition capability serves as a prerequisite for the ultimate “decoding” of the DNA program. By isolating candidate viral contigs, the tool prioritizes sequences for downstream functional analysis, such as the discovery of novel oncolytic viruses with therapeutic potential or the monitoring of emerging zoonotic threats. Thus, the framework does not merely categorize data but transforms raw sensor output into actionable biological intelligence, bridging the gap between high-throughput sequencing and real-time clinical decision-making.

Beyond traditional alignment tools like BLAST, profile-based methods such as HMMER3 have been widely used to detect remote viral homologies by leveraging hidden Markov models. While HMMER3 offers superior sensitivity in identifying conserved protein domains, it remains computationally intensive and depends on pre-defined multiple sequence alignments of known viral families. Conversely, our approach belongs to the alignment-free paradigm, which bypasses the need for reference templates or conserved domains. By extracting high-dimensional features directly from the raw sequence, our deep learning ensemble captures global genomic signatures that are often overlooked by both local alignment and profile-based methods, providing a faster and more generalized solution for the detection of highly divergent ‘unseen’ viruses.

To overcome the limitations of simple local alignment, more sophisticated iterative approaches like PSI-BLAST have been developed. PSI-BLAST enhances sensitivity by constructing position-specific scoring matrices (PSSMs) from initial hits, allowing for the detection of remote viral homologs. However, these methods are susceptible to ‘profile drift’, where the inclusion of non-homologous sequences in early iterations can lead to false-positive cascades. Furthermore, the iterative nature of PSI-BLAST is computationally demanding for high-throughput metagenomic sensing.

Our ensemble model provides a robust alternative by operating in a template-free manner. Unlike PSI-BLAST or HMMER3, which require an initial ‘anchor’ or a pre-defined profile to start the search, the deep learning architecture extracts features directly from the raw sequence. This allows for the identification of viruses in the ‘homology twilight zone’ without the computational overhead or the risk of profile corruption, even when the biological signal is obscured by 10% random noise.

## 4. Conclusions

In this study, we presented a convolutional neural network-based ensemble for the identification of viral sequences in heterogeneous human metagenomic samples. The proposed ensemble strategy combines complementary representations of DNA sequences, enabling the model to capture both local patterns and global distributional properties. Specifically, the architecture integrates multiple CNN configurations characterized by input features, different pooling mechanisms, and hyperparameter selection, which contribute distinct and complementary information to the final prediction. In conclusion, the proposed ensemble demonstrates strong robustness and generalization capabilities across multiple experimental settings, consistently outperforming existing state-of-the-art methods. Overall, this work provides both a high-performing solution and practical insights for future research on viral identification from raw DNA sequences. These findings reinforce the suitability of CNN-based models for viral genome analysis and highlight the importance of architectural diversity in achieving robust performance.

Future developments of this research will explore the integration of evolutionary information into the neural framework to further enhance the detection of novel pathogens. Specifically, we aim to investigate the use of Position-Specific Scoring Matrices (PSSMs), generated through iterative PSI-BLAST searches, as high-dimensional input features for the deep learning models. By combining the pattern recognition capabilities of convolutional neural networks with the sensitivity of profile-based evolutionary signatures, we expect to further improve the classification of ‘unseen’ viruses that reside in the remote homology twilight zone. This hybrid approach could provide a more robust diagnostic layer, bridging the gap between traditional sequence-alignment expertise and modern deep learning architectures in real-time metagenomic sensing. A further possible direction for future work is to integrate the Transformer-based model [7] used as a benchmark into our ensemble framework, with the goal of further improving performance. Additionally, it would be valuable to explore alternative sequence representation strategies beyond the one-hot encoding adopted in this study. In particular, representations incorporating physicochemical properties of nucleotides [39] or their interactions could provide richer and more biologically meaningful information to the model.

Finally, we provide a publicly available implementation of the proposed system to facilitate reproducibility https://github.com/LorisNanni/Ensemble-Deep-Learning-Models-on-Raw-DNA-Sequences-for-Viral-Genome-Identification-in-Human-Samples (accessed on 1 April 2026). The datasets are also available for download from the same link. 

## Figures and Tables

**Table 1:** Tested hyperparameter configurations.

Hyperparameter	Tested Configurations
Learning rate	0.1, 0.01, 0.001
Optimizer	Adam
Epochs	30
Batch size	128
Loss function	Binary Cross-Entropy
Dropout	0.1, 0.2, 0.5
Number of filters	1000, 1200, 1500
Kernel size	from 5 to 18
Norm type (only LP)	from 1 to 10

**Table 2:** Size of the evaluated datasets.

Dataset	Training Set	Validation Set	Test Set
VM [15]	211,239	26,405	26,405
Unseen	191,773	47,943	27,644
SIM	168,000	21,000	21,000
LongC [20]	25,263	8246	17,385

**Table 3:** Comparison with SOTA–AUROC and Confidence Interval (CI) on the VM dataset. Bold indicates the best performance.

Method (Year)	AUROC	CI
FullE (2026)	**0.939**	[0.929, 0.949]
M_*f*_+_*p*_+_*l*_ (2026)	0.935	[0.925, 0.945]
[21] (2018)	0.790	[0.773, 0.807]
VIR [15] (2019)	0.923	[0.911, 0.933]
6-mers [15] (2019)	0.875	[0.860, 0.890]
DVF [20] (2020)	0.913	[0.900, 0.924]
[7] (2023)	0.936	[0.926, 0.946]

**Table 4:** Comparison with SOTA–AUROC and Confidence Intervals (CI) on the Noise dataset. Bold indicates the best performance.

Method (Year)	1%	5%	10%	CI 1%	CI 5%	CI 10%
FullE (2026)	**0.937**	**0.922**	**0.892**	[0.926, 0.946]	[0.911, 0.933]	[0.879, 0.905]
M_*f*_+_*p*_+_*l*_ (2026)	0.932	0.915	0.883	[0.922, 0.942]	[0.903, 0.927]	[0.869, 0.897]
VIR [15] (2019)	0.921	0.902	0.863	[0.910, 0.933]	[0.890, 0.914]	[0.848, 0.878]
DVF [20] (2020)	0.909	0.885	0.843	[0.897, 0.921]	[0.872, 0.899]	[0.828, 0.859]

**Table 5:** Comparison with SOTA–AUROC and Confidence Interval (CI) on the Unseen dataset. Bold indicates the best performance.

Method (Year)	AUROC	CI
FullE (2026)	0.785	[0.775, 0.795]
M_*f*_+_*p*_+_*l*_ (2026)	**0.790**	[0.781, 0.800]
VIR [15] (2019)	0.761	[0.750, 0.772]
DVF [20] (2020)	0.757	[0.746, 0.768]

**Table 6:** Comparison with SOTA–AUROC and Confidence Interval (CI) on the LongC dataset. Bold indicates the best performance.

Method (Year)	AUROC	CI
FullE (2026)	**0.990**	[0.988, 0.992]
M_*f*_+_*p*_+_*l*_ (2026)	0.986	[0.983, 0.989]
VIR [15] (2019)	0.985	[0.982, 0.988]
DVF [20] (2020)	0.980	[0.975, 0.985]

## Data Availability

All datasets used in this study are publicly available on the GitHub repository associated with this paper.
